# ﻿ *Mazusjiangshiense* (Mazaceae), a new species from China: evidence from morphological and molecular analyses

**DOI:** 10.3897/phytokeys.241.117787

**Published:** 2024-04-09

**Authors:** Yong-Bin Chen, Xin-Yan Chen, Liang Ma, Zhuang Zhao, Shi-Pin Chen

**Affiliations:** 1 Fujian Vocational College of Bioengineering, Fuzhou, Fujian 350002, China Fujian Vocational College of Bioengineering Fuzhou China; 2 Sanming Garden Center, Sanming, Fujian 365000, China Sanming Garden Center Sanming China; 3 Fujian Health College, Fuzhou, Fujian 350101, China Fujian Health College Fuzhou China; 4 Key Laboratory of National Forestry and Grassland Administration for Orchid Conservation and Utilization at Fujian Agriculture and Forestry University, Fuzhou, Fujian 350002, China Key Laboratory of National Forestry and Grassland Administration for Orchid Conservation and Utilization at Fujian Agriculture and Forestry University Fuzhou China

**Keywords:** Lamiales, *Mazus*, molecular markers, morphology, nrITS, phylogenetics, *rbcL*, *rps16*, *trnL-trnF*

## Abstract

Utilising both morphological and molecular analyses, this study unveils *Mazusjiangshiense***sp. nov.**, a novel addition to the Mazaceae family, discovered in Shaowu County, Fujian Province, China. The comprehensive description and illustrations provided here are a result of a meticulous exploration of its morphological features. While bearing a resemblance to *M.gracilis*, this new-found species is distinguished by three distinct characteristics: its stems are delicately soft, its leaves possess a membranous quality and the ovary is notably villous at the apex. Integration of molecular evidence, derived from the nuclear ribosomal DNA (nrITS) and three plastid DNA sequences (*rps16*, *rbcL* and *trnL-trnF*), unequivocally supports the classification of *M.jiangshiense* as a distinct species. Notably, the molecular analysis positions it as a sister species to *M.spicatus*, underscoring the phylogenetic relationships within the genus *Mazus*. Our research not only introduces *M.jiangshiense* as a novel taxonomic entity, but also provides a nuanced understanding of its morphological differences and molecular affinities, enriching our comprehension of the diversity and evolutionary relationships of Mazaceae.

## ﻿Introduction

MazaceaeReveal (2011) stands as a recently established family within Lamiales, distinctively separated from Phrymaceae according to several phylogenetic studies (Albach et al. 2005; Xia et al. 2009; Schäferhoff et al. 2010). Comprising four genera and approximately 43 species (Xiang et al. 2021), *Mazus*Loureiro (1790) takes precedence as the largest genus, encompassing 39 accepted species primarily distributed in Asia and Australasia (Hong et al. 1998; Fischer 2004; Deng et al. 2016). In China, the Flora of China (Hong et al. 1998) originally recorded 25 species and three varieties, but subsequent reports introduced new species, such as *M.tainanensis* T.H.Hsieh (Hsieh 2000), *M.sunhangii* D.G.Zhang & T.Deng (Deng et al. 2016), *M.somggangensis* S.S. Ying (Ying 2019), *M.fruticosus* Bo Li, D.G.Zhang & C.L.Xiang (Xiang et al. 2021) and *M.danxiacola* Bo Li & B. Chen (Li et al. 2022). These additions suggest that China likely serves as the epicentre of distribution and diversity within *Mazus* (Yang 1979; Hsieh 2000). While investigating the background resources of protected areas in Fujian Province, south-eastern China, a distinctive *Mazus* species with a creeping stem and villous ovary was discovered. Through a meticulous analysis of flowering specimens and molecular data, it was unequivocally identified as a new species. Consequently, we hereby introduce and describe this novel entity as *Mazusjiangshiense* sp. nov.

## ﻿Materials and methods

In June and August of 2022, a comprehensive exploration of the putative new species was conducted within Jiangshi Provincial Nature Reserve, Shaowu City. Digital photographs of fresh plants in the field were taken by a Canon digital camera (6D) and flowers and fruits were collected and deposited in the Herbarium of the College of Forestry, Fujian Agriculture and Forestry University (FJFC). A Canon digital camera (6D) was used to document the plants in the laboratory, while micro-morphological features were examined under a stereomicroscope (SZM-41, LAOSVI).

To ascertain the phylogenetic position of this newly-discovered species, a curated set of 21 taxa within the *Mazus*, as per Li et al. (2022), was employed. Additionally, three species—*Dodartiaorientalis* Linnaeus (Linnaeus 1753), *Lanceatibetica* Hook.f. & Thomson (Hooker and Thomson 1857) and *Puchiumazuslanceifolius* (Hemsl.) Bo Li, D.G.Zhang & C.L.Xiang (Xiang et al 2021)—were chosen as outgroups. Detailed voucher information and GenBank accession numbers for all specimens are outlined in Table 1. The phylogenetic analyses were conducted employing three plastid DNA sequences (*rbcL*, *rps16* and *trnL-trnF*) and nuclear ribosomal ITS (nrITS). A total of 24 DNA sequences of *rps16*, 23 of *rbcL*, 23 of *trnL-trnF* and 20 of ITS were successfully obtained. By using the CTAB procedure of Doyle (1987), total genomic DNA was obtained from silica-dried leaves and the DNA was re-suspended in double-distilled water and kept at -40 °C. In a thermocycler (Eppendorf Scientific, Inc., Westbury, NY, USA), DNA amplifications were performed. For three plastid DNA sequences (rbcL, rps16 and trnL-trnF) and nuclear ribosomal ITS (nrITS), the fragments were respectively amplified with primers RH1/Z1352RC (Olmstead et al. 2001), rps16-2F/rps16-R3 (Bremer et al. 2002), c/f of Taberlet et al. (1991) and ITS1 and ITS4 (Wendel et al. 1995). There were 50 μl of final volumes used for PCR reactions, along with 1 μl of each specific primer (10 μM each), 5 μl 10× PCR buffer, 35–50 ng template, 1 μl 10 mM dNTP (2.5 mM each) and 0.2 μl MDBio Taq DNA Polymerase (MDBio, Taipei, Taiwan). After purifying PCR products with the Tian quick Midi Purification Kit according to the manufacturer’s instructions, they were sequenced directly. Sequences newly obtained have been deposited in GenBank. The evaluation of phylogenetic relationships utilised Maximum Parsimony (MP), Maximum Likelihood (ML), and Bayesian Inference (BI) techniques, executed on the CIPRES Science Gateway web server (RAxML-HPC2 on XSEDE v.8.2.12, PAUP on XSEDE v.4.a165 and MrBayes on XSEDE v.3.2.7a) as described by Miller et al. (2010), with parameters aligned as outlined by Chen et al. (2019).

**Table 1.** Taxon, vouchers and GenBank accession numbers of the specimens. The asterisk (*) indicates the sequences obtained in this study; the others are from GenBank. A dash (–) indicates missing data.

Taxon	Voucher	rbcL	rps16	trnL-trnF	ITS
*Mazusalpinus* Masam.	Sunhang11307 (Kun)	KX783481	KX783501	KX783520	MK192641
*M.caducifer Hance*	35025 (Kun)	KX783477	KX783497	KX783516	MK192664
*M.celsioides* Hand.-Mazz	KX783486 (Kun)	KX783486	MK266366	KX783525	–
*M.danxiacola* Bo Li & B. Chen 1	CB06425 (CSH)	ON323565	ON323567	ON323569	ON286711
*M.danxiacola* Bo Li & B. Chen 2	CB05735 (CSH)	ON323566	ON323568	ON323570	ON303604
*M.fauriei* Bonati	Sunhang11248 (Kun)	KX783479	KX783499	KX783518	LC034207
*M.gracilis* Hemsl.	–	FJ172729	FJ172701	FJ172687	FJ172738
*M.fruticosus* Bo Li, D.G. Zhang & C.L. Xiang	zdg4447 (Kun)	KX783470	KX783490	KX783509	MK192660
*M.humilis* Hand.-Mazz.	–	–	MK266367	MK266421	MK192667
*M.longipes* Bonati	Deng1941 (Kun)	KX783474	KX783494	KX783513	MK192652
*M.miquelii* Makino	–	MW238406	MW238406	MW238406	–
*M.novaezeelandiae* W.R. Barker	dtA68 (Kun)	KX783469	KX783489	KX783508	MK192676
*M.omeiensis* H.L. Li	nie1976 (Kun)	KX807209	KX807203	KX807208	MK192636
*M.procumbens* Hemsl.	zdg6074 (Kun)	KX783478	KX783498	KX783517	MK192647
*M.pulchellus* Hemsl.	dt093 (Kun)	KX783472	KX783492	KX783511	MK192638
*M.pumilio* R. Br.	Pagest.s.n.2021829 (Kun)	KX783468	KX783488	KX783507	MK192671
*M.pumilus* (Burm. f.) Steenis	–	FJ172728	FJ172700	FJ172686	FJ172737
*M.radicans* Cheesman	dt472 (Kun)	KT626738	MK266381	–	MK192635
*M.spicatus* Vaniot	–	FJ172730	FJ172703	FJ172689	FJ172740
*M.sunhangii* D.G. Zhang & T. Deng	zdg4142 (Kun)	KX783484	KX783504	KX783523	–
*M.xiuningensis* X.H. Guo & X.L. Liu	–	MW238409	MW238409	MW238409	–
*M.jiangshiense*	–	OP616018*	OP616019*	OP616020*	OP605381*
**Outgroup**					
*Puchiumazuslanceifolius* (Hemsl.) Bo Li, D.G.Zhang & C.L.Xiang	–	MW373737	MW373739	MW373741	MW364623
*Dodartiaorientalis* L.	XZ-2008-1	JQ342984	JQ342982	JQ342981	JQ342980
*Lanceatibetica* Hook.f. & Thomson	dt108 (Kun)	KX783467	KX807200	KX807205	MK192678

## ﻿Result and discussion

### ﻿Taxonomic treatment

#### ﻿ Mazus jiangshiense

Taxon classificationPlantaeLamialesMazaceae

Y.B. Chen, Xin Y. Chen & Liang Ma sp. nov.

255BAF14-0127-5EB9-B836-A01253C895FE

urn:lsid:ipni.org:names:77339920-1

##### Type.

China. Fujian Province, Shaowu County, 27°03'46"N, 117°15'40"E, elev. ca. 395 m, July 2022, *Y.B. Chen et al. 20220801015* (holotype: FJFC, POC592371!; isotype: CSH, POC592372!) (Figs 2, 3)

##### Diagnosis.

Results of morphological observation suggest that the newly-identified *Mazus* is similar to *M.gracilis* Hemsl. ex Forbes & Hemsl. (Forbes and Hemsley 1890) and *M.procumbens* Hemsl. ex Forbes & Hemsley (Forbes and Hemsley 1890), but it differs in the stem texture and whether the ovary is villous or not. It is also close to *M.spicatus* Vaniot (Vaniot 1905), but it can be easily distinguished by the growth habit of the stem and texture. Table 2 displays a detailed comparison of morphological characteristics.

**Table 2.** Comparisons amongst *M.jiangshiense* and morphologically similar species. A dash (–) indicates missing data.

Characteristic	*M.jiangshiense*	*M.gracilis*	*M.procumbens*	*M.spicatus*	*M.caducifer*	*M.radicans*
Plant	densely villous	glabrous or soon glabrescent.	white villous.	white to pale rusty villous.	white villous.	hirsute
Stem	creeping, soft, only inflorescence partially ascending.	creeping, hard, only inflorescence partially ascending.	creeping, hard, only inflorescence partially ascending.	erect or base sometimes tilted, hard, never creeping.	erect or ascending, hard.	creeping
Leaf	opposite or alternate, membranous, margin coarsely crenate, long petiolate, 2.5–5.5 cm.	opposite, herbaceous, margin crenate to subentire, short petiolate, 1–2.5 cm.	alternate or opposite, herbaceous, margin coarsely crenate, long petiolate, 1.5–6 cm.	opposite or upper ones alternate, membranous, margin incised-serrate, 1–4 cm.	opposite, papery, blade ovate-spatulate-petiolate, base tapering, margin coarsely irregularly serrate, 3.5–10 cm.	leaves few, entire, 2.5–3.5 cm.
Inflorescence	Racemes axillary or terminal, to 17.0 cm or more.	Racemes usually lateral, rarely terminal, ascending, to 15 cm.	Racemes terminal, to 13 cm or more.	Racemes terminal, to 20 cm.	Racemes terminal, to 35 cm.	flowers solitary.
Corolla	0.9–1.1 cm.	1.2–1.5 cm.	less than 1 cm.	0.8–1.2 cm.	1.3–1.8 cm.	–
Calyx	funnelform, 3.0–4.0 mm, lobes longer than tube.	campanulate, 4.0–7.0 mm, lobes as long as tube.	campanulate, ca. 5.0 mm, lobes as long as, or slightly longer than, tube.	campanulate, 5.0–8.0 mm in fruit, lobes as long as tube.	funnelform, ca. 1.3 cm in fruit, lobes almost as long as tube.	–
Ovary	long hirsute.	glabrous.	glabrous.	long, hirsute.	hirsute.	–

##### Description.

Perennials, densely villous. Stems creeping to 50 cm, soft, slender, branched, base woody, inter-node nearly 5 cm, often longer than or equal to leaves, nodal rooting. Basal leaves are several to numerous, often deciduous. Cauline leaves opposite or alternate, leaf blade subrounded or oblong, membranous, 2.5–5.5 cm long including petiole, 2.1–4.1 cm width, larger at base of the stem, adaxially green, abaxially greyish-green to silver grey, apex acute to obtuse, base truncate and tapering, margin coarsely crenate, both sides villous; lateral veins 3–4 pairs, fluted adaxially, elevated abaxially and conspicuous on both surfaces; petiole 0.5–2.5 cm, slender, villous. Racemes axillary or terminal, obliquely ascending, to 17.0 cm or more, villous, sparse, multiflowered to 20; pedicels slender, 0.6 cm in fruit, densely villous; Calyx funnelform, 3.0–4.0 mm, slightly enlarged in fruit, villous on both surfaces, lobes 5, lanceolate, acute and longer than the tube. Corolla light purple or white, 0.9–1.1 cm long, dotted yellow on the palate and with sparse glandular-hair, tube cylindrical with glandular-hair, 0.4–0.5 cm long; limb 2-lipped, upper lip bilobed, lobes lanceolate, apex acute, slightly upturned; lower lip trilobed, middle lobe oblong, smaller and narrower than lateral lobes, lateral lobes broadly ovate, spreading away from middle lobes; plaits with sparse glandular hairs; palate comprising 2 longitudinal elevations, mostly hidden in the corolla tube, covered by sparse glandular hairs, clavate hairs, white to transparent, 0.6 mm long. Stamens 4, filaments protruding from the tube, appressed to the corolla tube, glabrous, included, anterior pair longer, curved, anthers positioned adjacent to the corolla tube on upper lip; Ovary villous, styles ca. 0.5 cm long, stigma lamellate, included. Capsule oblong, ca. 2.5 mm long, ca. 1.5 mm diam., apex rounded and villous. Seeds brown-yellow, numerous.

##### Chinese vernacular name.

jiāng-shí-tōng-qúan-căo (将石通泉草).

##### Phenology.

The flowering period is from June to July and the fruiting period is from August to September.

##### Etymology.

The new species was named after the locality of Jiangshi Provincial Nature Reserve, where it was discovered.

##### Distribution and habitat.

The species is distributed in Xiao Jiafang Town of Shaowu County, northwest Fujian Province, China and grows under a stone cave in a cliff at an elevation of approximately 395 m (Fig. 2A).

##### Phylogenetic analysis.

**Figure 1.**
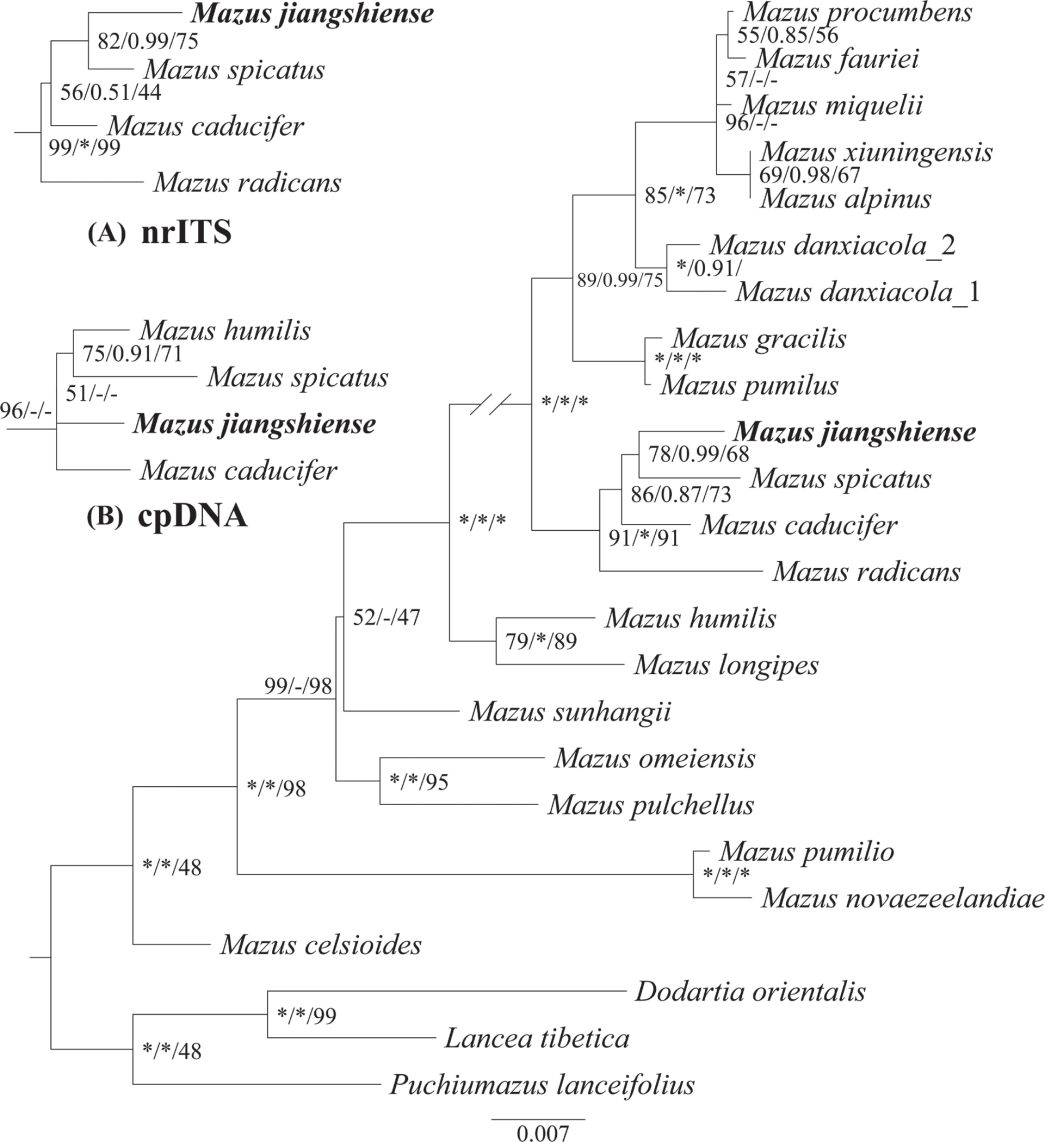
The phylogenetic tree was constructed by the plastid, ITS and combined matrix using Maximum Likelihood, Maximum Parsimony and Bayesian Inference methods. The numbers near the nodes are bootstrap percentages and Bayesian posterior probabilities (BP_ML_, PP, BP_MP_). Separate combined results of nrITS (**A**) and plastid (**B**) are shown in the top left corner. A dash (-) indicates that the node is inconsistent between the Bayesian tree and the topology of the MP/ML trees. The asterisk (*) node is 1.00 posterior probability or 100 bootstrap percentage.

The nucleotide sequence lengths for the new species nrITS, plastid *rps16*, *rbcL* and *trnL-trnF* are 550 bp, 806 bp, 1261 bp and 930 bp, respectively. Table 3 provides a summary of the characteristics of each dataset utilised in this study. The phylogenetic analyses are presented in the form of Maximum Likelihood (ML) trees and the support values, including bootstrap percentages (BS_ML_/BS_MP_) and posterior probabilities (PP), are indicated near the respective nodes. Upon analysis of the combined dataset, the phylogenetic trees consistently demonstrated that the new species is closely related to *M.spicatus*, garnering robust support (BP_ML_ = 78, PP = 0.99, BP_MP_ = 68). The tree derived from the nrITS dataset (Fig. 1A) corroborates this relationship, with strong support for the new species being the sister to *M.spicatus* (BP_ML_ = 82, PP = 0.99, BP_MP_ = 75). However, the plastid-based phylogenetic tree introduces an intriguing twist, indicating that the new *Mazus* species is proximate to *M.caducifer* Hance (Hance 1882), *M.spicatus* and *M.humilis* Hand-Mazz (Hong et al. 1998). This forms a collapsed topology (Fig. 1B), suggesting a potentially intricate evolutionary relationship amongst these species.

**Table 3.** Statistics from the three DNA datasets analysed.

DNA region	No. of taxa	Aligned length	No. variable characters (%)	No. informative characters (%)	Tree length	Consistency index	Retention index
ITS	20	608	178 (29.28)	125 (20.56)	303	0.75	0.83
Plastid	24	3069	257 (8.37)	130 (4.24)	320	0.87	0.90
Combined	24	3677	435 (11.83)	255 (6.94)	641	0.79	0.85

Conservation status. Following our comprehensive biodiversity survey of Shaowu Jiangshi Provincial Nature Reserve in July 2022, more than 500 individuals of *Mazusjiangshiense* have been identified in three distinct locations within the Reserve; the three populations are considerably distant from each other and collectively occupy an area of approximately 100 m^2^. Fruiting individuals were observed in each population. Given the management efforts of the Provincial Nature Reserve, it is currently at low risk of existential threats.

**Figure 2.**
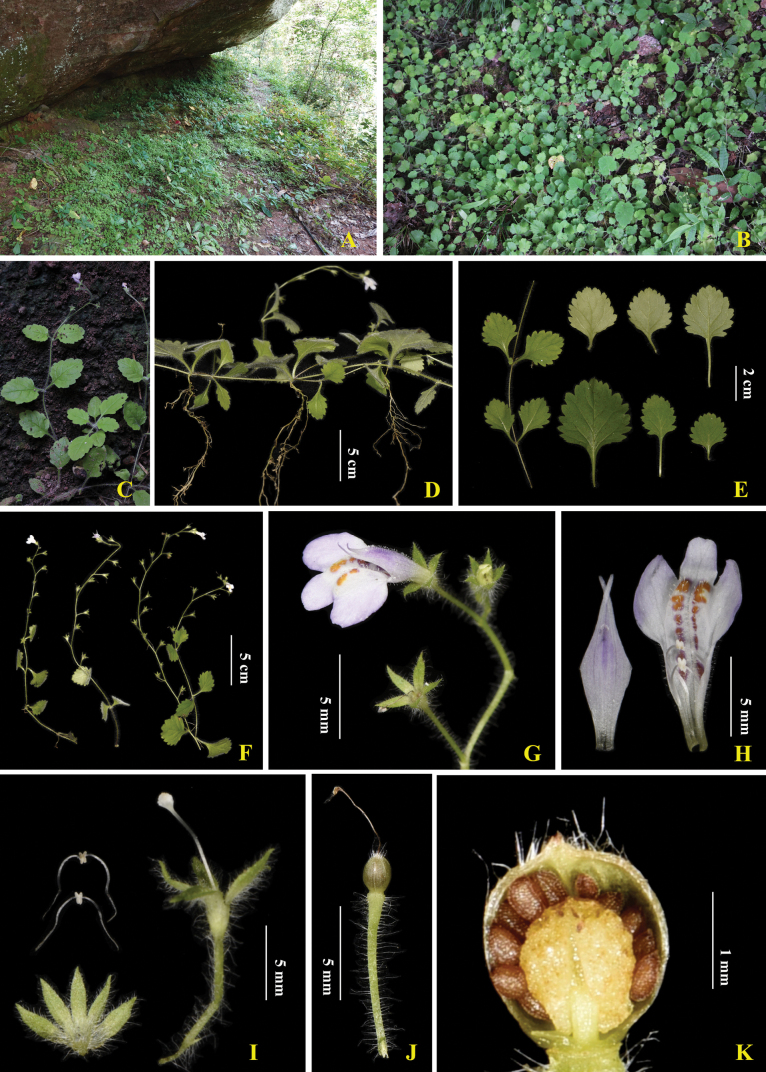
Morphology of *Mazusjiangshiense***A, B** habitat **C** flowering plant **D** roots and stolons **E** leaves **F** inflorescence **G** a mature inflorescence with flowers and fruits **H** corolla **I** pedicel, calyx, stamens and pistils **J** fruit **K** seed.

**Figure 3.**
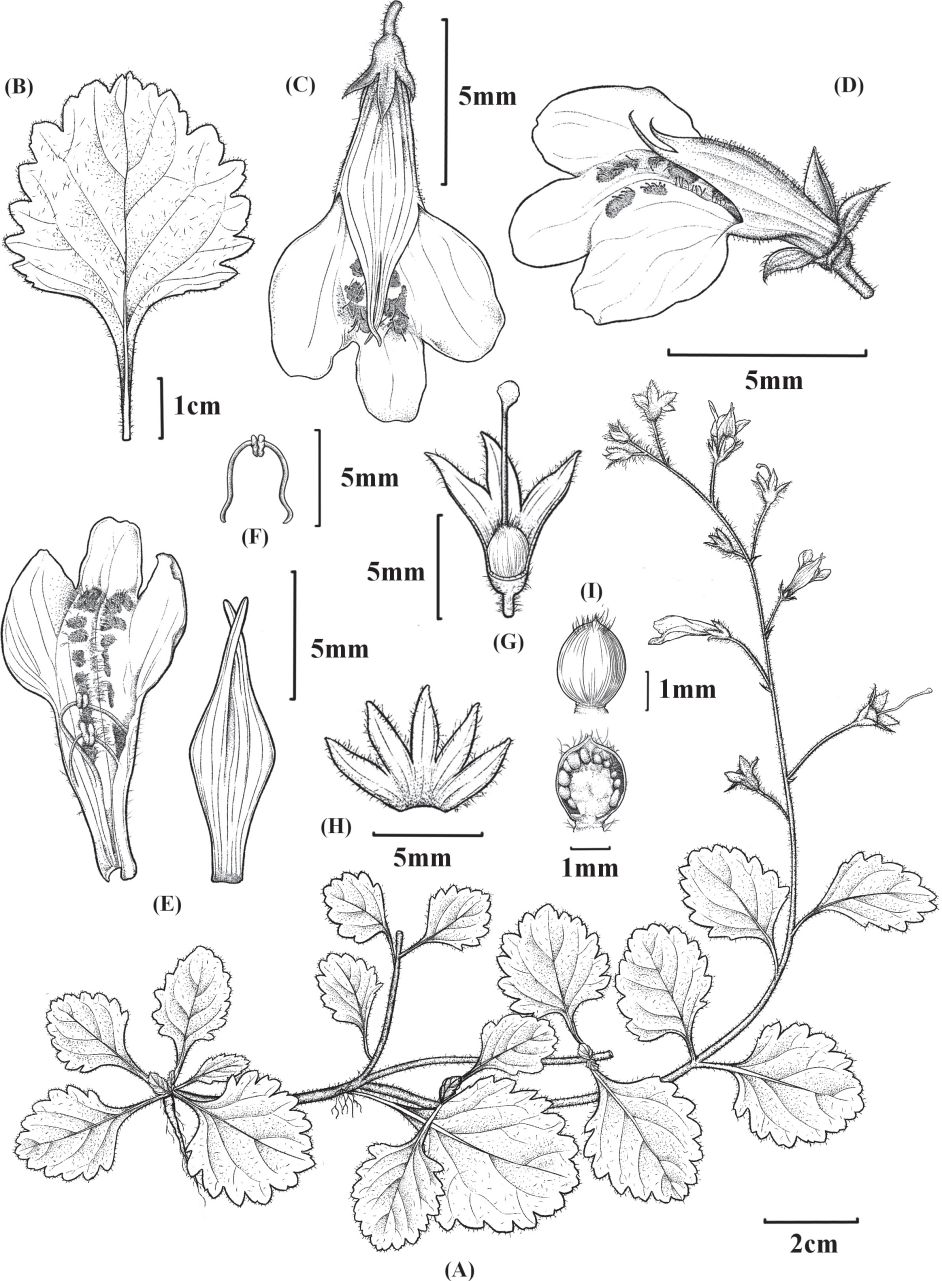
*Mazusjiangshiense* Y.B.Chen, Xin Y. Chen & Liang Ma **A** plant B leaves **C–E** corolla **F** stamens **G** pistils **H** calyx **I** seed.

## Supplementary Material

XML Treatment for Mazus jiangshiense

## ﻿Additional information

### Author ORCIDs

Yong-Bin Chen https://orcid.org/0000-0003-1099-3694

Xin-Yan Chen https://orcid.org/0000-0002-8632-2216

Liang Ma https://orcid.org/0000-0002-7366-7290

Zhuang Zhao https://orcid.org/0000-0002-8021-3879

Shi-Pin Chen https://orcid.org/0000-0002-8090-6616

### Data availability

All of the data that support the findings of this study are available in the main text.
